# Estimating the direct Covid-19 disability-adjusted life years impact on the Malta population for the first full year

**DOI:** 10.1186/s12889-021-11893-4

**Published:** 2021-10-09

**Authors:** Sarah Cuschieri, Neville Calleja, Brecht Devleesschauwer, Grant M. A. Wyper

**Affiliations:** 1 Department of Anatomy, Faculty of Medicine and Surgery, University of Malta, Msida, Malta; 2 Department of Public Health, Faculty of Medicine and Surgery, University of Malta, Msida, Malta; 3 Directorate for Health Information and Research, Ministry for Health, Gwardamangia, Malta; 4 Department of Epidemiology and Public Health, Sciensano, Brussels, Belgium; 5 Department of Veterinary Public Health and Food Safety, Ghent University, Merelbeke, Belgium; 6 Public Health Adviser, Place and Wellbeing Directorate, Public Health Scotland, Glasgow, Scotland

**Keywords:** Burden of disease, Disability-adjusted life years, DALY, YLD, YLL, Population health, Malta, European burden of disease network, Coronavirus, Covid-19

## Abstract

**Background:**

Disability-adjusted life years (DALYs) combine the impact of morbidity and mortality, allowing for comprehensive comparisons of the population. The aim was to estimate the DALYs due to Covid-19 in Malta (March 2020–21) and investigate its impact in relation to other causes of disease at a population level.

**Methods:**

Mortality and weekly hospital admission data were used to calculate DALYs, based on the European Burden of Disease Network consensus Covid-19 model. Covid-19 infection duration of 14 days was considered. Sensitivity analyses for different morbidity scenarios, including post-acute consequences were presented.

**Results:**

An estimated 70,421 people were infected (with and without symptoms) by Covid-19 in Malta (March 2020–1), out of which 1636 required hospitalisation and 331 deaths, contributing to 5478 DALYs. These DALYs positioned Covid-19 as the fourth leading cause of disease in Malta. Mortality contributed to 95% of DALYs, while post-acute consequences contributed to 60% of morbidity.

**Conclusions:**

Covid-19 over 1 year has impacted substantially the population health in Malta. Post-acute consequences are the leading morbidity factors that require urgent targeted action to ensure timely multidisciplinary care. It is recommended that DALY estimations in 2021 and beyond are calculated to assess the impact of vaccine roll-out and emergence of new variants.

## Background

The outbreak of novel coronavirus SARS-CoV-2 has been a global pandemic since early 2020 [1]. The small European state of Malta reported its first Covid-19 case on the 7th of March 2020 [2]. The first Covid-19 wave was well controlled in Malta, with a relatively low mortality rate and a total cumulative positive cases of 108 per 100,000 [3]. The second Covid-19 wave initiated in mid-Summer and this saw a high mortality and admission rates to hospital [4, 5]. The positivity and mortality rates were constantly high until mid-March 2021, where another instituted lockdown curbed the community spread [6]. Up till the end of March 2021, 6562 positive cases per 100,000 population have been reported with 88 deaths per 100,000 population [2].

Burden of disease assessments allow the estimation of a disease impact on a population by measuring the morbidity and mortality attribution and presenting it through a single metric called disability-adjusted life years (DALYs) [7]. Morbidity occurrence is translated through the estimation of years lived with disability (YLD) following the adjustment for the severity and the disability suffered due to a disease or injury. The mortality counts due to the disease or injury, are translated into estimates of years of life lost to premature mortality (YLL), using age-conditional life tables. DALYs are estimated by the combination of YLD and YLL [7]. This metric is a popular way used to assess the health impact at a population level and then translated as evidence to influence national policy makers.

The aim of this study was to estimate, for the first time, the direct impact of Covid-19 on the population of Malta for the first year (March 2020–2021). We also set to investigate the likely Covid-19 impact in relation to other causes of disease and injury, at a population level.

## Methods

### Data

Covid-19 mortality data between March 2020 and March 2021 were recorded from the daily Covid-19 bulletins issued by the Malta Ministry of Health [2]. However, mortality data did not differentiate from those dying with Covid-19 from those dying due to Covid-19, in line with WHO recommendations on assigning underlying cause of death [8]. Also, this group-level COVID-19 data did not provide details on the presence of any other underlying comorbidities among the infected individuals.

The Covid-19 positive cases were sub-divided according to the different health states, as shown in Table 1, based on the European Burden of Disease Network and the European Centre of Disease Prevention and Control (ECDC) Covid-19 consensus disease model [9]. Daily positive case numbers have been reported by the Malta Ministry of Health from the onset of the pandemic in Malta [2]. However, data on the different health states of the Covid-19 positive cases were provided on a weekly basis, every Friday, from the 7th of August 2020, following the initiation of the second wave in Malta [5]. Prior to this period, the Covid-19 situation was well controlled [3, 10]. Hence, weekly Covid-19 health states were not publicly provided except for the monthly intensive care unit (ICU – critical cases) admissions due to Covid-19 [11]. Therefore, estimations for the hospitalized Covid-19 cases (non-ICU, i.e., severe cases) from March to July 2020 were performed. An assumption was made that the proportion of Covid-19 admissions to hospital in August in relation to the total positive cases (not requiring ICU) was the same as that throughout March to July 2020. It was hence assumed that 1.3% of the Covid-19 cases not admitted to the ICU, required admission to a hospital ward i.e. were ‘severe’ cases.

**Table 1:** Covid-19 health states, descriptions and disability weights [9]

Health state	Description	Disability weight (95% uncertainty interval)
Asymptomatic	Has infection but does not experience any symptoms	Nil
Moderate (Community; seeking healthcare assistance)	Has symptoms and causing some difficulty with the daily activities. Does not require hospital admission.	0.051 (0.032–0.074)
Severe (Hospitalised; non-intensive care)	Has symptoms and causing great difficulty with daily activities.	0.133 (0.088–0.190)
Critical (Hospitalised; intensive care)	Intensive care admission with or without respiratory support	0.655 (0.579–0.727)
Post-acute consequences	Person still feels symptoms such as tired, fatigue, pain all over body, persisting after the acute infection	0.219 (0.148–0.308)

Considering that in Malta a high swabbing rate and efficient contact tracing, up to secondary contact, has been in effect from the onset of the pandemic, it was noted that 20% of the reported positive cases (not requiring hospitalisation, i.e., moderate cases) were actually asymptomatic [3, 5, 12]. Hence, for this study’s analyses, a reduction of 20% from the moderate cases was performed. However, the literature indicates that a proportion of Covid-19 will not be picked up through polymerase chain reaction (PCR) testing [13]. Indeed, the PCR sensitivity has been reported to be 97.2% [14]. Based on this, we have estimated that cases represent 80% of all infections, so have upscaled our estimates to reflect this to estimate the total number of asymptomatic and symptomatic infections. Additionally, it has been noted that approximately 20% of reported cases were actually asymptomatic [3, 5, 12]. To reflect this, we have also reduced the number of moderate cases by 20%, since by virtue of requiring healthcare assistance, any severe or critical cases would be symptomatic. Therefore, we estimated that asymptomatic cases represented a total of 36% of all cases as follows: (i) 20% of all cases are asymptomatic and not reported (ii) 20% of the reported cases (80%) are asymptomatic i.e. 16% of all cases.

### Analyses

The modelling approach applied by this study to calculate the Covid-19 DALYs was based on the European Burden of Disease Network and the ECDC consensus disease model [9].

YLL was estimated by combining the death counts by five-year age-groups and sex. The estimates were calculated by multiplying the number of deaths in each age-group by the age-conditional remaining life expectancy from the Global Burden of Disease (GBD) Study 2019 reference life table, where the same values are assigned to both males and females [15].

The symptomatic duration of 14 days was considered for each health state (critical, severe, moderate), following local and international reported durations of Covid-19 infection respectively [16, 17]. Person-years were calculated as the sum of cases for each symptomatic health states (critical, severe, moderate), multiplied by 14, divided by 365.25, reflecting the contribution of individual days to a complete year. This was carried out since DALYs use a year as the unit of time. An estimate of the post-acute consequence (long-Covid) YLD was calculated, where it was assumed that 13.3% (one in seven) of the total Covid-19 patients (symptomatic and asymptomatic) would suffer from long-Covid for a duration of 28 days [18]. The disability weights used for each health status was derived from the European Disability Weight Study (EDWS) and GBD 2019, as shown in Table 1 [19, 20].

YLD was estimated by multiplying person-years of infection with the disability weight for each health status (as per Table 1). The disability weight’s uncertainty levels (UI) were also considered to measure the upper and lower limits of the YLD. DALYs were then calculated through the summation of YLL and YLD. The upper and lower limits of the DALYs were also calculated based on the UIs of the YLD.

Our estimate of Covid-19 DALYs was compared to the 2019 leading ranked causes of disease and injury for Malta, using estimates from GBD 2019 [21].

### Scenario analyses

A number of uncertainties were present in our analyses especially when estimating YLD. The health state case numbers were not available prior to August 2020 (except for ICU on a monthly basis). When health state data was available, the cases were a snapshot of one particular day (Friday). Another uncertainty was the proportion of cases that suffered from post-acute Covid-19 consequences. Thus, in order to quantify the impact of these uncertainties, sensitivity analyses were performed. Sensitivity analyses were performed on the moderate cases and the post-acute consequences, since these were likely the most uncertain estimates. The sensitivity analyses assessed for the effect of an increase in moderate cases by 10, 25 and 50% on the DALYs. We also assessed the effect of different transition probabilities from an acute to post-acute consequence have on the DALYs, when considering all Covid-19 cases and then symptomatic cases only. Finally, different scenarios were assumed with half or double the duration of post-acute consequences and different proportions of moderate cases, in order to minimise and maximise the YLD.

## Results

Over the course of a year (March 2020 – March 2021), an estimated 70,421 people were infected by Covid-19 in Malta, both with and without symptoms. When considering the symptomatic cohort and taking into the account the duration of the Covid-19 infection; for an infection duration of 14 days, this reflected 1570 person-years. These generated a YLD of 93, as shown in Table 2. Out of the three health states, the moderate symptomatic cohort translated the most to the symptomatic YLD. Post-acute consequences were estimated to have affected 9366 patients with a contributing YLD of 157, if symptoms persisted for 28 days. Out of the different health states, post-acute consequences had the highest impact on YLD while the severe cases had the lowest impact on YLD. The mortality for this time period was of 331 deaths, contributing to 5229 YLLs.

**Table 2:** Measure of the population health impact of Covid-19

Health State	Morbidity			Mortality		DALYs (UI 95%)
	Persons	Person - years	YLD (UI 95%)	Deaths	YLL	
Covid-19 infection	70,421		250 (166–352)	331	5229	5478 (5395 - 5581)
Symptomatic Covid-19		1570	93 (60–131)			
Moderate		1507	77 (48–112)			
Severe		49	7 (4–9)			
Critical		14	9 (8–10)			
Post-acute consequences^a^	9366	718	157 (106–221)			

^a^duration of 28 days

It was estimated that the DALYs due to Covid-19 were 5478. The highest contributor factor to the DALYs was mortality, with YLL representing 95% of the DALYs with 16 YLL per Covid-19 death.

On comparing the DALYs for the top-ranking diseases and injuries as reported by GBD 2019 for Malta to this study’s estimated Covid-19 DALYs, it was noted that the Covid-19 DALY ranked fourth, after ischemic heart disease (13,594 DALYs), low back pain (7106 DALYs) and diabetes (5796 DALYs), but before stroke (5096 DALYs).

### Scenario analyses

A number of different sensitivity analyses, which varied assumptions on moderate cases (symptomatic community) and post-acute consequences health statuses were performed to evaluate their impact on DALYs, as shown in Table 3. Additionally, a combination of assumptions was performed to minimise and maximise the impact of YLD, with a range of 115 to 3606 (Table 3). The different defining scenarios involving the post-acute consequences and the combination assumption maximising the YLD, led to a higher estimated DALY than that estimated for Malta 2020. However, only when maximising the YLD, did the DALY represent a higher-ranking position for the cause of disease and disability for Malta, from initially placing fourth to second leading cause of disability.

**Table 3:** Covid-19 Morbidity sensitivity analyses with corresponding impact on YLD and DALYs, Malta, 2020

Impacted health status	Sensitivity	YLD (UI 95%)	DALYs (UI)
Moderate - Community cases	Increase by 10%	256 (170–361)	5484 (5399 - 5589)
	Increase by 25%	267 (178–377)	5495 (5406 - 5606)
	Increase by 50%	286 (190–405)	5514 (5418 - 5633)
Post-acute consequences for all cases	Transition probability of 26.6%	406 (272–571)	5634 (5501 - 5800)
	Transition probability of 6.65%	171 (113–241)	5399 (5342 - 5470)
	Duration of 56 days	406 (272–571)	5634 (5501 - 5800)
	Duration of 14 days	171 (114–241)	5399 (5342 - 5470)
Post-acute consequences for symptomatic cases only	Transition probability of 26.6%	275 (184–388)	5504 (5413 - 5617)
	Transition probability of 6.65%	138 (92–195)	5367 (5320 - 5424)
	Duration of 56 days	275 (184–388)	5504 (5413 - 5617)
	Duration of 14 days	138 (92–195)	5367 (5320 - 5424)
Combination to minimize the impact of YLD	No increse in Moderate case	115 (76–163)	5344 (5305 - 5392)
	Transition to post-acute consequences for symptomatic only		
	Half the transition of acute to post consequences (6.65%)		
	Half the duration for post-acute consequences (14 days)		
Combination to maximize the impact of YLD	Increase Moderate cases by 50%	3606 (2295 - 5201)	8834 (7524 - 10,429)
	Transition to post-acute for All cases		
	Double transition of acute to post consequences (26.6%)		
	Double duration for post-acute consequences (56 days)		

## Discussion

### Summary

DALYs due to Covid-19 in Malta were likely to have had the fourth largest population health impact relative to other diseases and injuries for 2020. The majority of the population health loss was due to mortality as it represented 95% of DALYs with 16 YLL per Covid-19 death. On comparing to the Scottish Covid-19 BoD study, our study’s mortality contributed to a lesser percentage to the DALYs than Scotland, where the latter reported mortality to contribute to 98% of DALYs [22].

In our study, morbidity was driven mostly by post-acute consequences, followed by symptomatic community cases (moderate). Indeed, when maximizing both post-acute consequences and moderate cases, the estimated DALY exceeded the impact of Covid-19 from fourth to the second leading cause of disability in Malta. Therefore, although Covid-19 individuals that require hospitalisation, with or without intensive care support, have a considerable ill-health impact, the post-acute consequences, also called “long-haulers” might pose the largest cumulative impact on the population health, although this follows a degree of uncertainty. This finding corresponds with the Scottish Covid-19 DALYs estimate although different time periods were considered in this study [23]. Nevertheless, it needs to be noted that following a comparison analyses between a Malta national burden of disease study for low back pain (LBP) to the GBD LBP estimates for Malta, it was concluded that the GBD overestimated the LPB burden [24]. Hence, it is likely that the Covid-19 DALYs estimated in this study may place at a higher-ranking position than fourth leading cause of disability in Malta.

### Strengths and weaknesses

The YLL calculation was based on the national reported cases (hospital, nursing homes and private residence deaths) that died while with Covid-19. Distinction was not made between those that died with Covid-19 or due to Covid-19. Since the onset of the pandemic, all deaths at hospital (even if initially negative for Covid-19), were tested for Covid-19 post-mortem however, deaths in private residents were not unless suspected by relatives that the deceased might have had Covid-19. This is more likely to have overestimated DALYs due to Covid-19, however this is counter-acted by the fact that deaths from early in the pandemic would have been less likely to have been identified as dying with Covid-19. The validity of the mortality estimate is furthermore supported by recent information on excess mortality. Compared to 2019, there have been 317 more deaths in 2020, which is in line with the number of reported Covid-19 deaths. Furthermore, caution needs to be put forward when comparing our study results to other Covid-19 burden of disease studies according to the mortality definition considered for the DALYs estimation.

The aspirational life table was used to calculate the YLL in order to facilitate comparative research considering that Covid-19 is a global issue, although acknowledge that this choice is an area of intense debate [25].

All admissions to hospital were tested for Covid-19, with PCR testing repeated if any symptoms develop during the patient’s stay or the patient needed to undergo an invasive procedure. Hence, the Covid-19 pick up rate for in-patient cases was efficient. However, hospital data was only freely available on a weekly basis from August. Although assumptions had to be made for this time period, acute YLD is very small in relation to the YLL and so the impact of these assumptions will be minimal. However, one cannot dismiss the possibility that the assumptions might have led to overestimations or underestimations for some cases.

At a community level, a high swabbing rate has been implemented from the very start of the pandemic in Malta with contact tracing up to the second contact of an infected person [3]. Therefore, Covid-19 positive cases, whether symptomatic or asymptomatic, were picked up at an early stage of the disease. Based on the literature it was assumed that the total Covid-19 population consisted of an additional 20% Covid-19 asymptomatic individuals that were never picked up [13]. However, this was an estimated proportion originating from the literature and does not necessarily reflect the local undetected Malta cases, hence we cannot exclude the possibility of overestimations or underestimations of this asymptomatic cohort.

The disease model to calculate the health loss due to Covid-19 morbidity was based on the European Burden of Disease Network and ECDC consensus method, which was adopted for both prevalence- or pathogen- based YLD calculations [9]. Meaning that any future presenting long-Covid conditions such as respiratory conditions, will be attributed to their relevant non-communicable diseases through prevalence data.

It needs to be noted that comorbidity data for the Covid-19 positive cases was not available. Common comorbidities such as diabetes and cardiovascular disease have been reported to increase the risk of acquiring Covid-19 infection (morbidity) as well as have a higher chance of mortality due to Covid-19 [26, 27]. However, burden of disease studies, including the Global Burden of Disease (GBD) Study, perform adjustments to downscale YLD to correct for multimorbidity. Indeed, it is uncommon for the YLL to be adjusted in such cases [28]. In terms of our study, since the major contribution to the DALYs was from the YLL rather than YLD, any adjustments to reduce the YLD would not have influenced our findings.

In our calculations, the post-acute consequences were based on assumptions since long Covid-19 remains largely uncertain and different entities provide different definitions and durations [29, 30]. Indeed, the scenario analyses were performed to try out a number of different assumptions and scenarios, while evaluating the corresponding DALYs. However, since the YLD contributed to only 5% of the DALYs, fluctuations in post-acute consequences had a greater impact on DALYs only when maximizing the YLD health status outcome. It is recommended that further research is performed on post-acute consequences to have a better understanding on the progress of this condition.

## Conclusions

Covid-19 has bestowed a substantial burden upon Malta’s population health. This study estimates the direct impact of Covid-19 in Malta and not the impact of lockdowns or implemented mitigation measures instituted throughout the duration of this pandemic. From our analyses it could be observed that Covid-19 presented as a novel disease in March 2020, however over 1 year it became the fourth leading cause of disability among the population. The burden of disease methodology used assessed for both the direct and indirect impacts of Covid-19. Indeed, it was clear that the largest morbidity impact of Covid-19 was arising from post-acute consequences. Although this is still an unknown territory, where even the definition is still debatable [29], it is clear that those that become ‘long haulers’ are attributing to higher DALY and inevitably will have a higher burden on the healthcare system. Indeed, more than 50 different symptoms have been reported to be experienced by these ‘long haulers’ with the commonest symptom being fatigue followed by headaches [31]. Therefore, it is recommended that further research on long Covid-19 is conducted at a national level. Additionally, long Covid-19 should be considered as an urgent public health matter with engagement of a multi-disciplinary service to provide targeted healthcare service to this sub-population.

Since the end of December 2020, Malta, has been administering Covid-19 vaccination to the population through a priority system [32]. Indeed, up to the time of writing (3rd May 2021), Malta has the fastest vaccination roll-out within the European Union (EU), with 25% of the total eligible population fully vaccinated and 54% having had the first dose [2]. Covid-19 vaccination is considered as the tool to tackle Covid-19 infection. Hence it is recommended that DALYs estimations are measured periodically during 2021 and beyond to determine the vaccines’ impact on the population, as well as determine the effect of emerging variants.

## Data Availability

The datasets used and/or analysed during the current study are all publicly available online on the Malta Ministry of Health website: https://www.facebook.com/sahhagovmt

## Abbreviations

Covid-19: Coronavirus Disease 2019
DALYs: Disability-Adjusted Life Years
ECDC: European Centre for Disease Prevention and Control
EDWS: European Disability Weight Study
GDB: Global Burden of Disease
ICU: Intensive Care Unit
YLD: Years Lived with Disability
YLL: Years of Life Lost to premature death
